# Mathematical and Algorithmic Advances in Machine Learning for Statistical Process Control: A Systematic Review

**DOI:** 10.3390/e28020151

**Published:** 2026-01-29

**Authors:** Yulong Qiao, Tingting Han, Zixing Wu, Ge Jin, Qian Zhang, Qin Xu

**Affiliations:** 1 School of Information Technology, Jiangsu Open University, Nanjing 210036, China; 2 School of Automotive Studies, Tongji University, Shanghai 201804, China; 3 State Key Laboratory of Space Power-Sources Technology, Shanghai Institute of Space Power-Sources, 2965 Dongchuan Road, Shanghai 200245, China; 4 School of Network Security, Jinling Institute of Technology, Nanjing 211169, China

**Keywords:** statistical process control, machine learning, high-dimensional data, autocorrelated time series, nonparametric thresholding, dimensionality reduction, imbalanced data, anomaly detection, federated learning, Industry 4.0

## Abstract

Integrating machine learning (ML) with Statistical Process Control (SPC) is important for Industry 4.0 environments. Contemporary manufacturing data exhibit high-dimensionality, autocorrelation, non-stationarity, and class imbalance, which challenge classical SPC assumptions. This systematic review, conducted following the PRISMA 2020 guidelines, provides a problem-driven synthesis that links these data challenges to corresponding methodological families in ML-based SPC. Specifically, we review approaches for (1) high-dimensional and redundant data (dimensionality reduction and feature selection), (2) autocorrelated and dynamic processes (time-series and state-space models), and (3) data scarcity and imbalance (cost-sensitive learning, generative modeling, and transfer learning). Nonlinearity is treated as a cross-cutting property within each category. For each, we outline the mathematical rationale of representative algorithms and illustrate their use with industrial examples. We also summarize open issues in interpretability, thresholding, and real-time deployment. This review offers structured guidance for selecting ML techniques suited to complex manufacturing data and for designing reliable online monitoring pipelines.

## 1. Introduction

### 1.1. The Evolution of Statistical Process Control

Statistical Process Control (SPC) provides the foundational statistical framework for monitoring, controlling, and improving manufacturing and service processes. Since the inception of Shewhart’s control charts, these methods have effectively reduced process variability and ensured product consistency [1]. Traditional techniques, such as Cumulative Sum (CUSUM) and Exponentially Weighted Moving Average (EWMA) charts, detect deviations from a stable state by assuming that process data are independent and identically distributed (i.i.d.) and follow a normal distribution [2]. This statistical approach has historically driven significant improvements in quality and efficiency across various sectors.

### 1.2. Complex Industrial Data in Industry 4.0

The integration of the Industrial Internet of Things (IIoT) and cyber-physical systems (CPS) within Industry 4.0 has significantly transformed industrial data characteristics [3]. Modern manufacturing environments utilize extensive sensor networks that generate high-frequency data streams. Although this massive volume of data offers potential for process optimization, it introduces structural complexities that challenge the assumptions of standard SPC [4]. This survey focuses on four critical data characteristics that define this modern landscape:

- High-dimensionality and Redundancy: Processes are now monitored by hundreds or even thousands of variables, including high-resolution images and spectral data. This leads to the “curse of dimensionality”, where traditional multivariate charts become insensitive and computationally expensive, while many variables are often highly correlated and redundant [5].
- Autocorrelation and Non-stationarity: High-frequency data collection inherently introduces strong temporal dependencies (autocorrelation) within the data streams. Furthermore, processes may exhibit non-stationary behavior due to tool wear, environmental changes, or shifting operational setpoints, violating the i.i.d. assumption central to classical SPC [6,7].
- Data Scarcity and Imbalance: While overall data volume is large, data corresponding to specific fault conditions or rare events are often scarce. This class imbalance makes it difficult for traditional models to learn effective representations for anomaly detection, a critical task in quality control [8,9].

In addition to these three structural challenges, Nonlinearity is a pervasive characteristic that cuts across all categories. The underlying physics of modern processes often involves complex, nonlinear interactions that traditional linear models fail to capture [10]. Recent studies have also emphasized the integration of mission reliability and risk-based maintenance into SPC frameworks to address operational risks such as product quality decline and production task delays [11,12]. Therefore, we treat nonlinearity not as a separate silo, but as a fundamental property addressed within each of the three main challenges.

### 1.3. Limitations of Classical SPC in Handling Complex Data

Faced with these data complexities, classical SPC methodologies often struggle to maintain robust performance. Shewhart-type charts are relatively insensitive to small or moderate process shifts, a limitation that is exacerbated in autocorrelated settings [13]. Although memory-based charts like EWMA and CUSUM improve sensitivity, their performance degrades when the i.i.d. assumption is violated [14]. Furthermore, multivariate charts such as Hotelling’s $T^{2}$ suffer from reduced sensitivity and interpretability issues in high-dimensional spaces [5]. Consequently, the mathematical framework of traditional SPC requires adaptation to handle the dynamic and complex data ecosystems of Industry 4.0, necessitating more advanced analytical approaches.

### 1.4. Research Gap and Contributions of This Survey

Machine learning (ML) has emerged as a powerful paradigm to address these limitations, offering a suite of algorithms capable of learning from complex data patterns. A growing body of literature has demonstrated the successful application of ML in SPC, as evidenced by numerous recent surveys [4,15,16]. However, these reviews often categorize the literature by ML technique (e.g., supervised, unsupervised) or by application area, providing a broad but fragmented overview. A critical research gap exists for a systematic, problem-driven survey that synthesizes and structures the field from a mathematical and algorithmic perspective, focusing on how specific ML methods address the specific challenges posed by complex industrial data.

To address this gap, we provide a comprehensive review of ML-based SPC techniques, organized by their capability to handle the data complexities outlined above. We adopt a problem-driven taxonomy that aligns complex data challenges in SPC with corresponding methodological families. Specifically, we (1) map high-dimensionality, autocorrelation, and data scarcity to classes of ML solutions; (2) explain the mathematical rationales that make these solutions effective for each challenge; and (3) synthesize open problems regarding interpretability, thresholding, and real-time scalability.

### 1.5. Scope and Organization of the Paper

This paper is structured to guide the reader from the foundational challenges to advanced solutions and future trends. Section 2 details the literature search methodology and presents our core conceptual framework, which classifies ML techniques based on the data challenges they address. Section 3, Section 4 and Section 5 form the technical core of the survey, systematically reviewing the mathematical and algorithmic approaches for handling high-dimensional data, autocorrelated processes, and scarce/imbalanced data, respectively. Each of these sections includes a discussion of key methodologies and relevant case studies. Section 6 provides a broader discussion, synthesizing the findings, and highlighting overarching challenges such as explainability, scalability, and real-time implementation. Finally, Section 7 concludes the paper by summarizing the key findings and delineating promising avenues for future research.

For the sake of mathematical clarity, we adopt a consistent notation throughout this manuscript: bold uppercase symbols (e.g., Σ) represent matrices, bold lowercase symbols (e.g., x) represent vectors, and italic symbols denote scalars.

## 2. Methodology and Systematic Literature Search

This systematic review was conducted following the Preferred Reporting Items for Systematic Reviews and Meta-Analyses (PRISMA) 2020 guidelines [17]. This section details the comprehensive and reproducible methodology, including the search strategy, study selection process, quality assessment of included studies, and the conceptual framework for classification.

### 2.1. Search Strategy and Databases

The literature search was performed on several major academic databases, including Scopus, Web of Science, IEEE Xplore, and Google Scholar. The search query was designed to capture the intersection of ML and SPC, using a combination of keywords and their synonyms. The primary search string was: ‘(“statistical process control” OR “SPC” OR “quality control”) AND (“machine learning” OR “deep learning” OR “artificial intelligence”) AND (“manufacturing” OR “industrial”)’.

To ensure comprehensive coverage of the specific data challenges addressed in this survey, this primary query was combined with specialized keyword groups corresponding to the core sections of our analysis:

- For Section 3 (High-Dimensionality): The query was augmented with terms such as ‘(“high dimensional” OR “multivariate” OR “image-based” OR “multi-sensor” OR “feature selection” OR “dimensionality reduction” OR “PCA” OR “autoencoder”)’.
- For Section 4 (Autocorrelation): We used additional keywords like ‘(“time series” OR “autocorrelated” OR “dynamic” OR “non-stationary” OR “LSTM” OR “recurrent neural network” OR “residual chart”)’.
- For Section 5 (Data Scarcity): The search included terms such as ‘(“imbalanced data” OR “rare event” OR “anomaly detection” OR “few-shot” OR “zero-shot” OR “transfer learning” OR “SMOTE” OR “generative adversarial network”)’.

The search was limited to articles published in English, with a primary focus on the period from 2014 to the present. This timeframe was selected to capture the explosive growth of deep learning (DL)applications in Industry 4.0, which has fundamentally reshaped SPC methodologies over the last decade. While this focus ensures coverage of state-of-the-art techniques, we acknowledge that some foundational pre-2014 works in traditional multivariate SPC may be less emphasized, though key seminal papers are included for context. This structured keyword strategy ensures that our review is both broad and deep, systematically covering the key methodological responses to modern data challenges in SPC.

### 2.2. Inclusion and Exclusion Criteria

Studies were included if they met the following criteria:

- The primary focus was on the application of one or more ML algorithms to an SPC problem.
- The context was related to industrial, manufacturing, or process monitoring.
- The study provided sufficient methodological detail or empirical results.

Exclusion criteria were:

- Articles where ML or SPC was only mentioned peripherally.
- Studies focused purely on traditional SPC methods without ML integration.
- Short conference abstracts or non-peer-reviewed articles.

### 2.3. Study Selection and PRISMA Flow

**Figure 1:**
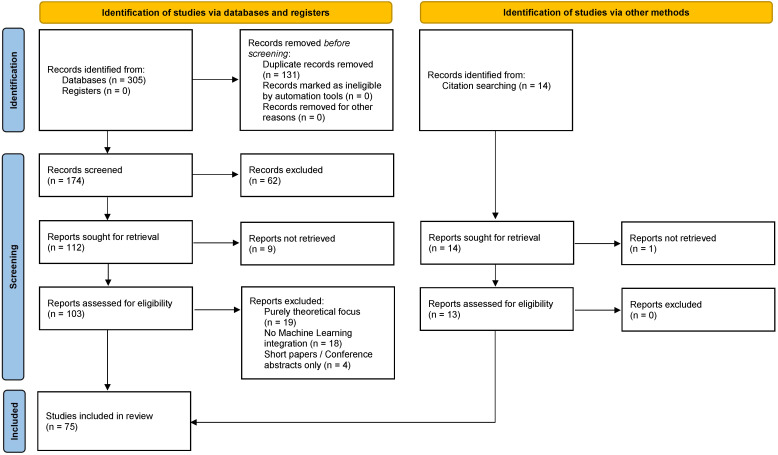
PRISMA 2020 flow diagram for new systematic reviews. The values presented are estimates based on the literature search (as of 1 December 2025).

The systematic search, performed on 1 December 2025, initially yielded 305 records from all queried databases. After the removal of 131 duplicate records, 174 unique records proceeded to title and abstract screening. Based on our predefined inclusion and exclusion criteria (Section 2.2), 62 records were excluded during this stage due to irrelevance (e.g., not related to SPC, ML peripheral, wrong domain). The remaining 112 full-text articles were sought for retrieval. Of these, 9 articles could not be retrieved, leaving 103 articles that were assessed for eligibility. Finally, 41 full-text articles were excluded after detailed review for reasons such as purely theoretical focus (19), no ML integration (18), and short papers/abstracts (4). In addition to the database search, 14 records were identified via citation searching. Of these, 1 report was not retrieved, and the remaining 13 were assessed as eligible and included. Consequently, a total of 75 studies were included in this systematic review. The complete study selection process is illustrated in the PRISMA 2020 flow diagram (Figure 1).

### 2.4. Quality Assessment of Included Studies

To ensure the reliability and validity of the findings synthesized in this review, a systematic quality assessment (QA) was performed on all included studies. Given the diverse nature of ML applications in SPC, a tailored QA checklist was developed, drawing inspiration from established guidelines for quality evaluation in engineering and computer science systematic reviews. Each included study was independently assessed by two reviewers (Y.Q. and T.H.) for the following criteria:

1. Clarity of Problem Definition: Was the specific SPC problem being addressed clearly defined, along with the industrial context?
2. Methodological Rigor and Transparency: Was the ML algorithm and its integration with SPC described in sufficient detail to allow for replication? Were the experimental setup, data sources, and evaluation metrics clearly stated?
3. Validation and Generalizability: Was the proposed methodology validated using appropriate datasets (e.g., real-world industrial data or realistic simulations)? Were the results discussed in terms of their practical implications and generalizability?

Discrepancies between reviewers were resolved through discussion and, if necessary, consultation with a third reviewer (Q.X.). Studies were categorized based on their adherence to these quality criteria, with higher quality studies given greater weight in the synthesis of findings. This systematic QA process enhances the trustworthiness of our review’s conclusions.

### 2.5. A Taxonomy of Complex Data Challenges in SPC

**Figure 2:**
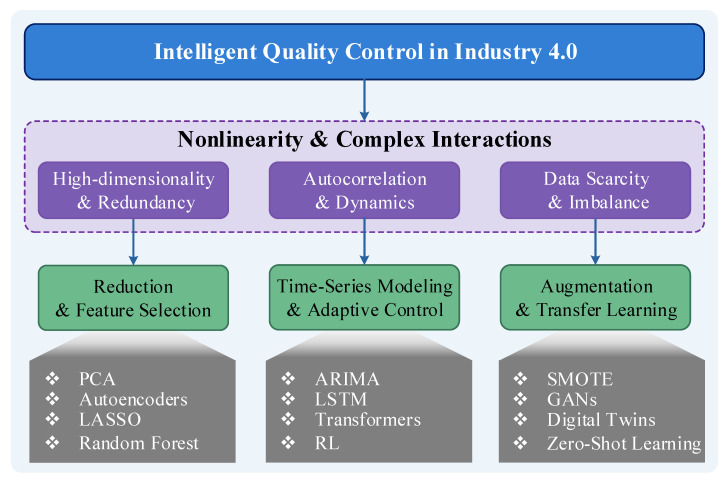
A Framework of Complex Data Challenges and Corresponding ML Solutions in SPC. This figure illustrates the mapping from high-level manufacturing goals to specific data challenges (e.g., high-dimensionality, autocorrelation) and the corresponding classes of ML methodologies (e.g., dimensionality reduction, time-series models) used to address them.

From the selected literature, we derive a taxonomy that structures the field based on the problem domain. Instead of a technology-centric view, we adopt a challenge-centric perspective. As will be detailed in the subsequent sections, we classify the ML-driven SPC methodologies based on their primary capability to address a specific type of data complexity. This problem-driven taxonomy, illustrated conceptually in Figure 2, forms the organizational backbone of this survey and provides a clear framework for understanding the role of different algorithms.

## 3. Methodologies for High-Dimensional and Redundant Data

### 3.1. Mathematical Challenge: The “Curse of Dimensionality” in Process Monitoring

In modern manufacturing, processes are often monitored by a vast number of variables, stemming from multiple sensors, high-resolution imaging systems, or spectral measurements. This high-dimensional data space (p≫n, where *p* is the number of variables and *n* is the number of observations) presents a formidable challenge known as the “curse of dimensionality.” For traditional multivariate SPC charts like the Hotelling’s $T^{2}$, the covariance matrix becomes difficult to estimate accurately and may be singular, leading to poor performance and high false alarm rates [5]. Furthermore, many of these variables are highly correlated, introducing multicollinearity and redundancy that can obscure the true sources of process variation. Recent comprehensive reviews have highlighted the shift from traditional approaches to advanced high-dimensional monitoring techniques that address these specific challenges [18]. The core mathematical challenge, therefore, is to transform the high-dimensional raw data into a lower-dimensional, more informative representation without losing critical information about the process state.

From a mathematical viewpoint, as the number of variables *p* approaches or exceeds the number of observations *n*, the sample covariance matrix $\hat{\Sigma }$ required for the classical Hotelling’s $T^{2}$ statistic becomes ill-conditioned or singular. This singularity renders the standard $T^{2}$ calculation numerically unstable, leading to inflated false alarm rates. To mitigate this, Principal Component Analysis (PCA) projects the observation x onto a lower-dimensional subspace spanned by the first *k* principal components. Letting $t=P^{T}\left(x-\bar{x}\right)$ be the *k*-dimensional score vector, monitoring is performed via a decomposition into the systematic variation in the latent space ($T_{\mathrm{PCA}}^{2}$) and the residual variation (*Q*):

(1) $$T_{\mathrm{PCA}}^{2}=t^{T}\Lambda_{k}^{-1}t,\;Q={∥x-\bar{x}-Pt∥}_{2}^{2},$$

where $\Lambda_{k}$ is the diagonal matrix of the first *k* largest eigenvalues. This separation allows for stable monitoring even when p≫n, provided that the process variability is well-captured by the first *k* components. However, a key challenge remains in interpreting these latent signals; a fault detected in the reduced space may not be immediately traceable to a specific physical sensor without additional diagnostic tools like contribution plots.

### 3.2. Feature Extraction and Dimensionality Reduction

Feature extraction methods aim to project the original data onto a lower-dimensional space by creating new features that are combinations of the original ones. These techniques are fundamental to modern multivariate SPC.

#### 3.2.1. Linear Methods

PCA is arguably the most widely used linear technique. It identifies orthogonal directions (principal components) that capture the maximum variance in the data. By retaining the first few principal components, one can effectively reduce dimensionality while preserving most of the process information. The monitoring is then performed on the scores of the retained components and the squared prediction error (SPE) in the residual space [19]. Partial Least Squares (PLS) is another popular method, particularly useful when there is a specific quality output variable, as it seeks to find latent variables that maximize the covariance between the process variables and the output. For instance, Bamdad [20] utilized Principal Component Regression (a variant of PCA) for sustainable environmental monitoring in metal processing, demonstrating the utility of linear methods in reducing complex data to manageable indices. However, traditional linear projections often struggle with the “curse of dimensionality” in two-sample tests. To address this, advanced tests such as the Srivastava and Du (SD) and Bai and Saranadasa (BS) tests have been adapted into control charts (e.g., SDEWMA, BSEWMA) to detect small shifts in high-dimensional streams more effectively than standard $T^{2}$ charts [21]. Other works, such as Yao et al. [22], have also successfully applied PCA for feature extraction in sensor networks. The principles of multivariate monitoring are further extended in various specialized control charts, such as those for the multivariate coefficient of variation [23] and dual multivariate CUSUM charts [24], which, while not always ML-based, establish the foundation for handling multiple variables.

#### 3.2.2. Nonlinear Methods

When process relationships are nonlinear, linear methods like PCA are insufficient. Nonlinear dimensionality reduction techniques have thus gained prominence. Kernel PCA (KPCA) extends PCA by first mapping the data into a high-dimensional feature space via a kernel function, where linear separation is then possible. Recent advancements have introduced efficient variants like Reduced Kernel PCA-based spectral clustering to mitigate the computational burden of KPCA in large-scale industrial datasets [25]. Autoencoders (AE), a type of neural network, offer a powerful and flexible framework for learning nonlinear data representations. An AE is trained to reconstruct its input, forcing it to learn a compressed representation (the “bottleneck” layer) of the data. Variational Autoencoders (VAEs) extend this by learning a probabilistic distribution of the latent space, which can be useful for generative tasks and uncertainty quantification [26]. Mathematically, an AE consists of an encoder h(·) and a decoder g(·). The monitoring statistic is typically the Reconstruction Error (RE), defined as the squared Euclidean distance between the input x and the reconstruction $\hat{x}=g\left(h\left(x\right)\right)$:

(2) $$\mathrm{RE}\left(x\right)={∥x-\hat{x}∥}_{2}^{2}={∥x-g\left(h\left(x\right)\right)∥}_{2}^{2}.$$

This metric serves as a nonlinear counterpart to the *Q*-statistic in PCA. These DL approaches are central to handling complex, high-dimensional data such as images. For example, Marconato et al. [27] proposed a framework to extract human-interpretable semantic concepts from image data in manufacturing, while McKinney et al. [28] used unsupervised fusion to compress multimodal sensor data into a low-dimensional space for monitoring additive manufacturing. The Partitioning Ensemble Control Chart (PECC) proposed by Yeganeh et al. [5] also leverages ensemble learning on partitioned image data, effectively managing high dimensionality. This is particularly relevant for modern applications like monitoring in wire arc additive manufacturing [29] and robust fault monitoring in automotive systems [30].

### 3.3. Feature Selection and Regularization

In contrast to feature extraction, feature selection aims to identify a subset of the original variables that are most relevant to the process monitoring task, discarding redundant or irrelevant ones. This not only improves computational efficiency but also enhances model interpretability, as the monitoring is based on original, physically meaningful variables. Regularization methods, such as the Least Absolute Shrinkage and Selection Operator (LASSO), can perform automatic feature selection by shrinking the coefficients of less important variables to exactly zero. Tree-based ensemble methods like Random Forest can also be used for feature selection by ranking variables based on their contribution to the model’s predictive accuracy (e.g., Gini importance). Wu et al. [31] proposed a nonparametric monitoring scheme for high-dimensional processes based on Random Forest, showcasing its dual capability for monitoring and feature selection. The integration of such ML models (including Support Vector Regression (SVR), Feedforward Neural Network (FFNN), Random Forest (RF), eXtreme Gradient Boosting (XGBoost)) into control chart frameworks has been shown to improve shift detection across various data distributions [32]. These techniques are crucial for building parsimonious and robust monitoring models in high-dimensional settings, as seen in applications ranging from food production [33] to network surveillance [34].

### 3.4. Tree-Based and Graph-Based Monitoring Approaches

Beyond vector-based representations, recent advances have introduced methods capable of handling more complex data structures.

#### 3.4.1. Isolation Forests for Anomaly Detection

For high-dimensional data where defining a “normal” boundary is difficult, the Isolation Forest (iForest) algorithm offers a unique non-parametric approach. Unlike distance-based or density-based methods that scale poorly with dimensionality, iForest explicitly isolates anomalies rather than profiling normal points. It relies on the principle that anomalies are “few and different”, making them easier to isolate in a random tree structure (requiring fewer splits) compared to normal points. Wang and Liu [35] developed a new multivariate control chart based on the isolation forest algorithm, demonstrating its superior computational efficiency and robustness in high-dimensional settings compared to traditional $T^{2}$ charts.

#### 3.4.2. Graph Neural Networks

In many industrial systems, such as sensor networks or distributed manufacturing lines, the variables have an underlying topological structure (e.g., physical connectivity or causal relationships). Graph Neural Networks (GNNs) are designed to capture these dependencies directly. By representing sensors as nodes and their interactions as edges, GNNs can learn complex topological dependencies and spatial correlations that are invisible to standard multivariate models. Li et al. [36] utilized GNNs to model the interdependencies in sensor networks, significantly improving fault detection rates in interconnected systems by effectively reducing the high-dimensional sensor data into informative graph embeddings. Similarly, Bao et al. [37] proposed a dynamic graph embedding PCA framework that integrates temporal and spatial correlations, demonstrating superior fault detection performance in chemical and hot rolling processes. This graph-based perspective represents a frontier in high-dimensional SPC, moving from treating variables as a flat vector to understanding them as a structured system. However, a trade-off exists between topological awareness and computational feasibility. Graph construction and GNN inference are significantly more computationally intensive than linear projections like PCA. In high-speed manufacturing contexts where cycle times are measured in milliseconds, this latency can be prohibitive, underscoring the need for research into lightweight graph architectures or specialized hardware acceleration for real-time deployment.

### 3.5. Establishing Control Limits: Non-Parametric Thresholding

A critical yet often overlooked mathematical challenge in ML-based SPC is the determination of control limits. Traditional SPC relies on the assumption of normality to set limits (e.g., μ±3σ). However, the monitoring statistics derived from ML models, such as the reconstruction error (RE) of an Autoencoder or the anomaly score of an Isolation Forest, rarely follow a Gaussian distribution. They are often skewed and heavy-tailed. Consequently, applying standard 3σ limits leads to inaccurate Type I and Type II error rates. To address this, non-parametric thresholding techniques are essential. Kernel Density Estimation (KDE) is frequently used to estimate the probability density function of the monitoring statistic from the in-control validation data. The control limit CL is set as the (1−α)-th quantile of the empirical distribution estimated from in-control validation data:

(3) $$\int_{-\infty }^{CL}{\hat{f}}_{\mathrm{KDE}}\left(x\right)\;dx=1-\alpha ,$$

where α is the desired false alarm rate. For non-negative monitoring statistics (e.g., reconstruction error), using 0 as the lower bound is equivalent. Alternatively, bootstrapping methods or extreme value theory (EVT) can be employed to estimate the tail distribution more accurately for setting robust thresholds [31]. It is worth noting that the choice of the kernel function and bandwidth in KDE significantly impacts the sensitivity of the control limits. While Gaussian kernels are standard, cross-validation methods (e.g., likelihood cross-validation) should be rigorously applied to select the optimal bandwidth to avoid over-smoothing (masking faults) or under-smoothing (inflating false alarms). Similarly, in LASSO-based feature selection, the regularization parameter λ must be tuned via cross-validation to balance model sparsity and predictive accuracy [31].

## 4. Methodologies for Autocorrelated and Dynamic Processes

### 4.1. Mathematical Challenge: Violation of the i.i.d. Assumption

One of the most fundamental assumptions in classical SPC is that process observations are i.i.d. random variables. However, with the advent of high-frequency data acquisition in Industry 4.0, this assumption is frequently violated. Data streams from modern sensors are often characterized by strong serial correlation (autocorrelation), where the current observation is dependent on past observations. Applying standard SPC charts directly to autocorrelated data leads to a severely inflated false alarm rate, as the charts misinterpret natural process dynamics as special cause variation [13]. Furthermore, many industrial processes are inherently non-stationary and dynamic, with characteristics that evolve over time due to factors like tool wear, raw material changes, or environmental fluctuations. The mathematical challenge is to develop monitoring schemes that can effectively distinguish between common cause autocorrelation and genuine out-of-control signals, while adapting to the dynamic nature of the process.

To illustrate the impact of autocorrelation, consider a simple autoregressive process of order one, AR(1),

(4) $$X_{t}=\phi X_{t-1}+\varepsilon_{t},\;\varepsilon_{t}∼N\left(0,\sigma^{2}\right),$$

with |ϕ|<1. Even if the noise terms $\left\{\varepsilon_{t}\right\}$ are i.i.d., the resulting sequence $\left\{X_{t}\right\}$ exhibits serial dependence. The variance of the sample mean ${\bar{X}}_{n}$ no longer scales as $\sigma^{2}/n$, but is inflated by a factor depending on ϕ, leading to underestimation of the true variability when classical control limits, derived under independence, are used. Quantitatively, under an AR(1) process $X_{t}=\phi X_{t-1}+\varepsilon_{t}$ with |ϕ|<1 and $\varepsilon_{t}∼N\left(0,\sigma^{2}\right)$, the variance of the sample mean inflates as

(5) $$\mathrm{Var}\left({\bar{X}}_{n}\right)=\frac{\sigma^{2}}{n(1-\phi^{2})}\left[1+2\sum_{k=1}^{n-1}\left(1-\frac{k}{n}\right)\phi^{k}\right],$$

which for large *n* is well-approximated by $\mathrm{Var}\left({\bar{X}}_{n}\right)\approx \frac{\sigma^{2}}{n{\left(1-\phi \right)}^{2}}$. This derivation highlights the physical implication of autocorrelation: positive autocorrelation (ϕ>0) reduces the effective sample size, leading to an underestimation of the process variance if standard formulas are used. This underestimation narrows the control limits erroneously, resulting in frequent false alarms even when the process is in control.

### 4.2. Time-Series Forecasting and Residual Analysis

A primary strategy for handling autocorrelation is the “residuals approach.” This involves fitting a suitable time-series model to the process data to capture its predictable, autocorrelated structure. The control chart is then applied to the model’s residuals (the difference between the observed and predicted values), which should, in theory, be approximately i.i.d.

In the residual-based framework, one first fits a time-series model such as an Autoregressive Integrated Moving Average (ARIMA) model, denoted ARIMA(p,d,q), to the in-control data. Denoting the backshift operator by *B*, an ARIMA model can be written in the compact form

(6) $$\phi \left(B\right){\left(1-B\right)}^{d}X_{t}=\theta \left(B\right)\varepsilon_{t},$$

where $\phi \left(B\right)=1-\phi_{1}B-\cdots -\phi_{p}B^{p}$ and $\theta \left(B\right)=1+\theta_{1}B+\cdots +\theta_{q}B^{q}$. After estimating the parameters ${\hat{\phi }}_{i}$ and ${\hat{\theta }}_{j}$, one computes the one-step-ahead forecast ${\hat{X}}_{t|t-1}$ and the residuals

(7) $$e_{t}=X_{t}-{\hat{X}}_{t|t-1}.$$

Under a correctly specified model and in-control conditions, the residual sequence $\left\{e_{t}\right\}$ should be approximately i.i.d. with zero mean and constant variance. A Shewhart or EWMA chart is then applied to $\left\{e_{t}\right\}$, effectively restoring the distributional assumptions required by classical SPC. In deployment, residual diagnostics (e.g., Ljung–Box tests for whiteness and checks for stability/normality) should be performed on $\left\{e_{t}\right\}$, and model parameters updated on a rolling window to accommodate non-stationarity.

#### 4.2.1. Time-Series Models

The ARIMA model is a classical time-series forecasting method that has been widely used in this context. An appropriate ARIMA model is first identified and fitted to the in-control data, and then a Shewhart or EWMA chart is applied to the one-step-ahead forecast errors [6]. While effective for linear processes, ARIMA models struggle to capture complex nonlinear dynamics. Spatio-temporal data streams add another layer of complexity, which recent works have started to address by modeling both temporal and spatial correlations simultaneously [38,39].

#### 4.2.2. Recurrent Neural Networks and Variants

To address nonlinearity, Recurrent Neural Networks (RNNs) and their more advanced variants, such as Long Short-Term Memory (LSTM) and Gated Recurrent Unit (GRU) networks, have become the methods of choice. These networks are explicitly designed with feedback loops, allowing them to maintain an internal state or “memory” of past information. This makes them exceptionally well-suited for modeling sequential and time-dependent data. For instance, Chen and Yu [40] developed a residual control chart based on a deep RNN to monitor autocorrelated processes. Yeganeh et al. [7] demonstrated the superiority of an LSTM-based control chart for monitoring bivariate autocorrelated processes in the automotive industry. Similarly, Khaldi et al. [41] provided a comparative study on the best RNN-cell structures for different time-series behaviors. The attention-based Convolutional LSTM (ConvLSTM) autoencoder proposed by Tayeh et al. [42] further enhances this capability by allowing the model to focus on the most salient parts of the time series for anomaly detection. Despite their effectiveness, deep recurrent architectures introduce specific deployment challenges. Unlike ARIMA models, which are parameter-efficient and relatively interpretable, LSTM-based models are data-hungry and prone to overfitting if the training phase lacks sufficient temporal diversity. Furthermore, the opaque nature of the hidden state transitions complicates the root cause analysis, often requiring auxiliary explainability modules to be practically useful for operators.

### 4.3. State-Space Models and Adaptive Control

An alternative to residual-based methods is to model the process dynamics directly using state-space representations and adaptive algorithms. These methods are particularly powerful for non-stationary processes where the underlying dynamics may change over time. Kernel-based methods, for example, can model complex, nonlinear processes. Kim and Lee [10] proposed a kernel-based composite control chart for nonlinear and heteroscedastic time series, which can adapt to changing volatility. Reinforcement Learning (RL) also offers a powerful framework for adaptive control, where an agent learns an optimal policy to keep the process in a desired state by interacting with the environment. This approach is inherently suited for dynamic optimization [43,44]. Furthermore, adaptive control charts, which adjust their parameters (like control limits or sampling intervals) based on real-time process conditions, are gaining traction. For example, Zaman and Khan [45] developed an adaptive CUSUM chart using supervised learning to dynamically monitor process parameters. More recently, Bayesian frameworks have been integrated with adaptive EWMA charts to track complex quality profiles, using hybrid score functions to enhance sensitivity to small shifts while quantifying uncertainty via posterior distributions [46]. Abbas et al. [47] proposed a risk-adjusted EWMA chart that adapts to shifting patient risk profiles in healthcare, a principle directly transferable to industrial processes with changing risk states [14].

### 4.4. Spatio-Temporal Process Monitoring

A growing class of industrial data, such as thermal imaging of additive manufacturing processes or distributed sensor networks, exhibits both spatial and temporal correlations. Traditional methods that treat these dimensions separately fail to capture the complex evolution of the process. Zhou et al. [39] introduced an ML-based control chart specifically designed for spatio-temporal data streams. Leveraging the feature extraction capabilities discussed in Section 3, these approaches integrate spatial encoders (e.g., Convolutional Neural Networks (CNNs) or GNNs) with temporal modeling (e.g., LSTM). In this hybrid framework, the spatial model handles the high-dimensionality at each time step, while the temporal model captures the dynamics of the resulting latent vectors, allowing for the detection of local anomalies that evolve over time. This capability is critical for processes like 3D printing where a defect in one layer propagates to subsequent layers.

## 5. Methodologies for Data Scarcity and Imbalance

### 5.1. Mathematical Challenge: Learning from Few or Unrepresentative Samples

A common paradox in industrial data is the simultaneous abundance of normal operation data and the scarcity of fault data. Process failures and quality deviations are, by design, rare events. This leads to a severe class imbalance problem, where the number of “in-control” samples vastly outweighs the “out-of-control” samples. From a mathematical standpoint, this imbalance poses a significant challenge to many ML algorithms, which tend to develop a bias towards the majority class, resulting in poor detection performance for the rare but critical fault conditions [8]. Furthermore, when introducing new products or processes, there is often a “cold start” problem with very limited historical data available for any class. The challenge is to develop models that can learn effective decision boundaries from imbalanced datasets or generalize from limited experience.

From the perspective of statistical learning, class imbalance can be formalized as a mismatch between empirical class frequencies and the desired decision costs. Consider a binary classifier $f_{\theta }\left(x\right)$ trained with a logistic loss

(8) $$L\left(\theta \right)=\frac{1}{n}\sum_{i=1}^{n}\ell \left(y_{i},f_{\theta }\left(x_{i}\right)\right),\;\ell \left(y,f\right)=\log\left(1+\exp(-yf)\right),$$

where $y_{i}\in \left\{-1,+1\right\}$. In highly imbalanced SPC data, the majority class dominates this empirical risk, leading to a decision boundary biased towards the in-control class. A common remedy is to introduce class-dependent weights $w_{+},w_{-}$:

(9) $$L_{w}\left(\theta \right)=\frac{1}{n}\sum_{i=1}^{n}w_{y_{i}}\;\ell \left(y_{i},f_{\theta }\left(x_{i}\right)\right),$$

where $w_{+}>w_{-}$ compensates for the scarcity of the fault class. In DL-based SPC, the focal loss further down-weights well-classified samples and focuses the optimization on hard, typically minority, examples. For a predicted probability $p_{t}$ of the true class, the focal loss is defined as

(10) $$\ell_{\mathrm{focal}}\left(p_{t}\right)=-\alpha {\left(1-p_{t}\right)}^{\gamma }\log\left(p_{t}\right),$$

with tunable parameters α∈(0,1] and γ≥0. In practice, $p_{t}$ can be obtained from the margin via a sigmoid mapping, $p_{t}=\sigma \;\left(y\cdot f_{\theta }\left(x\right)\right)$, ensuring consistency between margin-based and probability-based formulations. These cost-sensitive formulations provide a principled way to counteract imbalance at the loss level, complementing data-level techniques such as SMOTE and GAN-based augmentation. In addition to these, emerging strategies such as ranked-set sampling [48] and hybrid sampling algorithms (combining ADASYN with edited nearest neighbors) [49] have shown promise in improving the efficiency and sensitivity of monitoring schemes without necessarily increasing the data volume requirements.

### 5.2. Data Augmentation and Generative Models

One direct way to combat data imbalance is to augment the dataset by creating synthetic samples of the minority class.

#### 5.2.1. Over-Sampling Techniques

The Synthetic Minority Over-sampling Technique (SMOTE) is a widely adopted method that creates “synthetic” minority samples by interpolating between existing minority samples in the feature space. Chu et al. [8] demonstrated that combining data feature enhancement, including SMOTE with smoothing techniques, with ensemble learning can significantly improve the recognition of control chart patterns.

#### 5.2.2. Generative Adversarial Networks

A more powerful approach for data augmentation is offered by Generative Adversarial Networks (GANs). A GAN consists of two neural networks, a Generator and a Discriminator, that are trained in a competitive setting. The Generator learns to create realistic data samples, while the Discriminator learns to distinguish between real and generated samples. Once trained, the Generator can produce high-fidelity synthetic fault data to balance the training set, enabling more robust classifier training. This generative capability is invaluable in SPC for simulating a wide range of potential process deviations. Zhang et al. [50] provides a valuable benchmark dataset for unsupervised anomaly detection, which inherently involves imbalanced classes and can be used to validate such generative approaches. Other techniques, such as removing representation bias through dataset resampling, also contribute to mitigating this issue [51]. Nevertheless, reliance on generative models carries the risk of “hallucination”, where synthetic samples may violate physical constraints or thermodynamic properties of the actual process. If a classifier overfits to these unrealistic artifacts, detection performance in the real world will degrade. Consequently, incorporating physics-informed constraints into the loss functions of GANs is becoming a critical quality gate for synthetic data generation in industrial contexts.

### 5.3. Digital Twins for Synthetic Data Generation

Beyond purely data-driven generative models, Digital Twins (DT) offer a physics-based approach to data augmentation. A Digital Twin is a high-fidelity virtual counterpart of a physical system that integrates multi-physics simulations with real-time data. In the context of data scarcity, DTs can be used to simulate rare failure modes and extreme operating conditions that are dangerous or expensive to reproduce in the real world. By running simulations under these varied conditions, massive amounts of labeled “synthetic” fault data can be generated to train ML models. dos Santos et al. [52] reviewed the use of simulation models in creating digital twins for production logistics, highlighting their potential in generating training data for predictive maintenance. Mattera et al. [29] also demonstrated the integration of physical knowledge with data-driven models (Hybrid Digital Twins) to monitor wire arc additive manufacturing, effectively overcoming the lack of experimental data for specific defect types. This convergence of physics-based simulation and ML represents a robust solution to the small data problem in high-stakes manufacturing.

### 5.4. Knowledge Transfer and Low-Shot Learning Paradigms

When data for a specific task is scarce, leveraging knowledge from related but different tasks or domains can be highly effective. This is the core idea behind transfer learning.

#### 5.4.1. Domain Adaptation via Transfer Learning

In the context of SPC, a model pre-trained on a data-rich production line can be fine-tuned for a new, data-scarce line. This approach, successfully demonstrated by Mih et al. [53] for automated defect detection in smart manufacturing, significantly reduces the amount of labeled data required to achieve high performance. The underlying assumption is that different but related processes share common underlying feature representations that can be transferred. Lin et al. [44] also explores Sim2Real transfer, where a model trained in a data-rich simulation is transferred to a real-world control task, which is a promising direction for overcoming the limitations of physical data collection.

#### 5.4.2. Strategies for Minimal Data: Zero-Shot and Few-Shot Learning

Zero-Shot Learning (ZSL) and Few-Shot Learning (FSL) represent the frontier of learning with limited data. ZSL aims to classify instances of classes that were not seen during training, typically by learning a mapping between visual features and semantic attributes. Li et al. [9] pioneered a ZSL-based approach for concurrent control chart pattern recognition, enabling the identification of new pattern types without retraining. FSL, meanwhile, focuses on learning from a very small number of examples. Similarity-based frameworks, as explored by Aburakhia et al. [54] for predictive maintenance, often underpin these methods, where a new sample is classified based on its similarity to a few known support samples. These techniques, along with weak supervision strategies [55], hold immense promise for agile manufacturing environments where new products and potential fault types emerge frequently. Specifically for small-sample sequence monitoring, transfer learning can be combined with self-starting control charts. Shang et al. [56] proposed a method for small-sample Poisson profiles that transfers knowledge from similar processes to estimate parameters for a new process, effectively mitigating the cold-start problem and approximating the optimal detection performance of classical charts even with limited baseline data. Similarly, Bayesian network models based on gray correlation have been developed to diagnose fault sources in multistage manufacturing systems with limited data [57].

## 6. Discussion and Open Challenges

Our review, structured around the key data challenges in modern SPC, reveals a clear trend: the field is moving from applying generic ML models to developing highly specialized algorithms tailored to specific data complexities. To provide a consolidated view of the state of the art, Table 1 synthesizes the key methodologies discussed in Section 3, Section 4 and Section 5. It maps each data challenge to its corresponding algorithmic solutions, highlighting their respective strengths, limitations, and representative literature. This comparison serves as a quick reference guide for selecting appropriate techniques based on the specific characteristics of the industrial data at hand.

However, the synthesis of these methodologies also highlights several overarching challenges and opportunities that will shape the future of data-driven quality control.

### 6.1. Synthesis of Findings: The Power of Hybridization

A recurring theme across all categories is the power of hybrid approaches. No single ML model is a panacea. The most effective solutions often combine techniques to address multiple facets of a problem. For example, a common and powerful pipeline involves first using a nonlinear dimensionality reduction technique like an Autoencoder to handle high-dimensionality, followed by applying an LSTM network to the lower-dimensional latent space to model temporal dynamics [29]. Similarly, generative models (GANs) can be used to create synthetic data to train a robust supervised classifier, thus tackling both data scarcity and classification simultaneously. The integration of ML models with traditional control charts, such as in EWMA-SVR hybrids [58] or adaptive CUSUM-SVR systems [45], further exemplifies this trend, blending the predictive power of ML with the statistical rigor of SPC. Future research should focus more on these synergistic frameworks, exploring optimal ways to combine feature engineering, dynamic modeling, and adaptive learning into cohesive end-to-end solutions.

### 6.2. Explainability for Trustworthy SPC

Despite the superior performance of many DL models, their “black-box” nature remains a major barrier to adoption in high-stakes industrial settings. Process engineers and quality managers need to understand why a model flags a deviation, not just that it has occurred. This has spurred the growth of explainable artificial intelligence (XAI) in manufacturing [59,60]. While our survey did not focus on XAI as a primary category, it is a critical cross-cutting challenge. Future ML-based SPC systems must integrate interpretability by design. This could involve:

- Using inherently interpretable models (e.g., decision trees, linear models) where possible, even if it involves a slight trade-off in performance [61].
- Employing post-hoc explanation techniques (e.g., SHapley Additive exPlanations (SHAP), Local Interpretable Model-agnostic Explanations (LIME)) to provide feature importance scores for black-box model predictions [62].
- Developing novel neuro-symbolic approaches that combine the learning power of neural networks with the logical reasoning of symbolic AI [63].

### 6.3. Comparative Analysis of Nonlinear Frameworks

As highlighted throughout this review, nonlinearity is a cross-cutting challenge addressed by various methodologies. While each section focused on specific data types, it is beneficial to compare these approaches holistically. Kernel methods (e.g., KPCA [10,64]) offer a mathematically rigorous way to handle nonlinearity by mapping data to a high-dimensional Hilbert space. They are particularly effective when the sample size is moderate, and the nonlinear manifold is smooth. However, they scale poorly with *n* due to the Gram matrix computation ($O(n^{3})$), as noted in [25]. In contrast, DL approaches (e.g., Autoencoders, LSTMs [7]) approximate nonlinear functions through hierarchical feature learning. They scale better with large datasets and can capture highly complex, non-smooth topologies (e.g., in image data). Their primary drawback is the risk of overfitting when data is scarce and the lack of interpretability. Manifold learning techniques (e.g., Isomap, LLE) occupy a middle ground, preserving local geometry, but are often sensitive to noise and outliers common in industrial data. Future research should focus on hybrid frameworks that combine the robustness of kernel methods with the scalability of DL, such as Deep Kernel Learning.

### 6.4. Limitations of Current ML-Based SPC

Despite the promising advances, practical limitations remain. A significant concern is the risk of overfitting, particularly in DL models trained on scarce or imbalanced data. Models that achieve near-perfect detection on training sets often fail to generalize to new operating conditions or unseen fault types. While techniques like dropout, regularization, and few-shot learning [9] mitigate this, rigorous validation on external datasets is often lacking in the literature. Furthermore, the computational efficiency of complex ensembles (e.g., Random Forests [31]) or deep recurrent networks can be prohibitive for millisecond-level real-time control. Robustness to label noise is another critical gap; industrial datasets often contain mislabeled events, which can severely degrade the performance of supervised learners.

### 6.5. Scalability and Real-Time Implementation

The transition from offline model development to real-time, online deployment presents significant engineering and algorithmic challenges. The computational cost of many advanced models can be prohibitive for real-time monitoring on the factory floor. Key research directions include:

- Edge Computing (EC): Pushing model inference to edge devices closer to the data source to reduce latency and network bandwidth requirements [65,66]. This requires the development of lightweight, efficient models (e.g., through quantization or knowledge distillation [67]).
- Distributed and Federated Learning (FL): For large-scale, multi-factory settings, distributed learning frameworks are essential. FL, in particular, offers a privacy-preserving approach where models are trained locally on distributed data without the need to centralize sensitive information, which is crucial when dealing with non-i.i.d. data across different sites [68,69].
- Efficient Algorithms: Continued research into more efficient optimization algorithms and model architectures is crucial for reducing the computational footprint of ML in SPC [70,71,72].

Digital Twin frameworks also present a pathway to integrate physics-based modeling with real-time ML control, enabling time-series process optimization even in complex additive manufacturing scenarios [73].

### 6.6. Human-in-the-Loop: Synergizing ML with Domain Expertise

Finally, it is crucial to recognize that the goal of ML in SPC is not to replace human experts but to augment their capabilities. Effective systems will incorporate a “human-in-the-loop” philosophy, where the ML model provides data-driven insights and recommendations, but the final decision-making authority rests with the process engineer. This requires intuitive user interfaces, interactive visualizations, and models that can seamlessly integrate domain knowledge provided by experts [74,75].

## 7. Conclusions and Future Prospects

In this survey, we have systematically reviewed the landscape of ML applications in SPC, framed through the lens of modern complex industrial data challenges. By moving beyond a technology-centric classification, we have structured the field around the core problems of high-dimensionality, autocorrelation, and data scarcity, providing a problem-driven roadmap for researchers and practitioners. Our analysis shows a clear maturation of the field, from applying off-the-shelf ML algorithms to developing sophisticated, often hybrid, methodologies tailored to the specific mathematical and statistical properties of industrial data.

The field is transitioning from generic ML applications to problem-driven, often hybrid, SPC systems that respect the statistical properties of industrial data. Future research must bridge the gap between algorithmic sophistication and shop-floor reality. We propose the following specific research directions:

1. Unified Hybrid Architectures: How can we optimally combine dimensionality reduction (e.g., AEs) with temporal modeling (e.g., LSTMs) and non-parametric thresholding into a single, end-to-end differentiable pipeline? Research should focus on joint optimization of these components rather than sequential training.
2. Physics-Informed Machine Learning (PIML): Moving beyond purely data-driven models, how can physical laws (e.g., thermodynamics in additive manufacturing) be incorporated into the loss functions of ML models? This is critical to ensure that synthetic data generated by GANs and anomaly scores are physically consistent and interpretable.
3. Trustworthy and Explainable Deployment: How can we quantify the uncertainty of ML-based control charts in real time? Research is needed into probabilistic DL methods (e.g., Bayesian Neural Networks) that provide confidence intervals alongside anomaly scores, fostering operator trust.
4. Dynamic Adaptation and Lifelong Learning: How can monitoring systems update their control limits in real time to accommodate natural process aging (drift) without catastrophic forgetting of previous fault signatures? Developing lightweight online learning algorithms for edge deployment is a key technical pathway.

Addressing these challenges will pave the way for resilient and autonomous quality control systems in the future.

For practitioners, this survey provides a guide for diagnosing their specific data challenges and selecting appropriate classes of ML solutions. For researchers, it highlights gaps and opportunities in interpretability, thresholding, and scalable deployment under non-stationarity.

## Figures and Tables

**Table 1:** Comparison of ML-based SPC Methodologies for Different Data Complexities.

Data Challenge	Methodology	Key Algorithms & References	Strengths	Limitations
High-Dimensionality & Redundancy	Dimensionality Reduction; Feature Selection; Graph Learning	PCA/PLS [20]; AE/VAE [27,28]; Random Forest [31]; GNN [36]; Isolation Forest [35]	Handles multicollinearity; Extracts latent patterns; Robust to noise; Captures topological dependencies (GNN).	Loss of physical interpretability (AE); Linear assumptions fail on manifolds (PCA); Computationally intensive.
Autocorrelation & Dynamic Processes	Residual Analysis; Time-Series Forecasting; Adaptive Control	ARIMA [6]; LSTM/GRU/RNN [7,40]; Reinforcement Learning (RL) [44]; Adaptive CUSUM [45]; Kernel Methods [10]	Models temporal dependencies; Reduces false alarms; Adapts to non-stationarity and dynamic shifts.	Training complexity (RNN); Model selection difficulty (ARIMA); Data hungry (RL); “Black-box” nature.
Data Scarcity & Imbalance	Data Augmentation; Generative Models; Transfer Learning	SMOTE [8]; GANs [50]; Digital Twins [29]; Transfer Learning [53]; Zero/Few-Shot Learning [9]	Balances class distribution; Enables cold-start monitoring; Generates diverse synthetic scenarios.	Synthetic data fidelity issues; Mode collapse (GANs); Risk of negative transfer; Simulation–reality gap.

## Data Availability

Data is contained within the article.
